# Native lignin extraction from soft- and hardwood by green and benign sub/supercritical fluid extraction methodologies†

**DOI:** 10.1039/d3ra01873c

**Published:** 2023-07-20

**Authors:** Federica Nardella, Jens Prothmann, Margareta Sandahl, Peter Spégel, Erika Ribechini, Charlotta Turner

**Affiliations:** a Department of Chemistry and Industrial Chemistry, University of Pisa Via G. Moruzzi 13 56124 Pisa Italy; b Lund University, Department of Chemistry, Centre for Analysis and Synthesis Lund Sweden Charlotta.Turner@chem.lu.se

## Abstract

Lignin constitutes an impressive resource of high-value low molecular weight compounds. However, robust methods for isolation of the extractable fraction from lignocellulose are yet to be established. In this study, supercritical fluid extraction (SFE) and CO_2_-expanded liquid extraction (CXLE) were employed to extract lignin from softwood and hardwood chips. Ethanol, acetone, and ethyl lactate were investigated as green organic co-solvents in the extractions. Additionally, the effects of temperature, CO_2_ percentage and the water content of the co-solvent were investigated using a design of experiment approach employing full factorial designs. Ethyl lactate and acetone provided the highest gravimetric yields. The water content in the extraction mixture had the main impact on the amount of extractable lignin monomers (LMs) and lignin oligomers (LOs) while the type of organic solvent was of minor importance. The most effective extraction was achieved by using a combination of liquid CO_2_/acetone/water (10/72/18, v/v/v) at 60 °C, 350 bar, 30 min and 2 mL min^−1^ flow rate. The optimized method provided detection of 13 LMs and 6 lignin dimers (LDs) from the hardwood chips. The results demonstrate the potential of supercritical fluids and green solvents in the field of mild and bening lignin extraction from wood.

## Abstract 2

A mild and green extraction method enabling recovery of native lignin from wood chips using sub/supercritical carbon dioxide mixed with water-modified ethanol, acetone or ethyl lactate as solvent.

## Graphical abstract

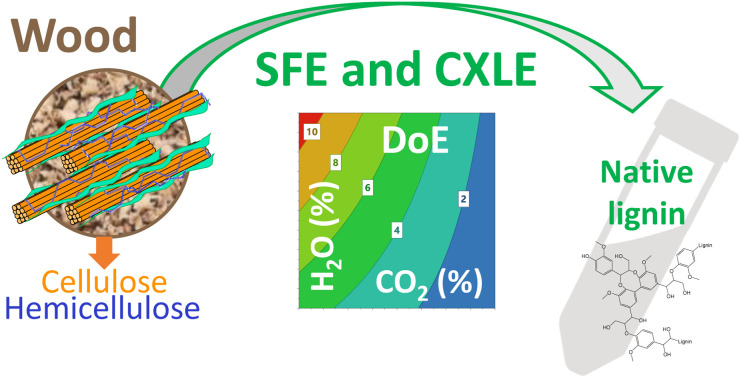

## Introduction

Lignocellulosic biomass has become of great interest in the past few years for its potential to provide sustainable fuels and valuable chemicals.^1–3^ Lignin, a major component of lignocellulosic biomass, is a biopolymer based on *p*-coumaryl alcohol (H-unit), coniferyl alcohol (G-unit), and sinapyl alcohol (S-unit).^4,5^ It is usually produced as a byproduct of the wood pulping process, and most of the obtained lignin is directly burnt to recover energy.^6^ Lignin is the most abundant renewable source of aromatic compounds, but methods capable of producing value from this biopolymer are currently underdeveloped. The chemical structure of lignin is still not completely resolved and depends on species and geographic origin.^7,8^ Due to its complexity, heterogeneity and reactivity, it has been difficult to isolate lignin in its native form without introducing chemical modification.^9,10^ Hence, lignin extraction from biomass is a key process^11,12^ to provide high-value compounds and its chemical characterization is of fundamental importance.^13,14^

Currently, the main methods used for lignin isolation are based on either lignin desolvation or hydrolysis of the polysaccharides, leaving lignin as an insoluble residue.^15,16^ In the Kraft and organosolv processes, the separation of lignin from the polysaccharide fraction is performed using basic and acidic conditions, respectively. On the contrary, in the Klason process the polysaccharides are hydrolysed by sulphuric acid and the lignin is left as a solid residue.^17^ However, these processes require high temperatures and pressures, as well as long reaction times and therefore producing unwanted side-reactions as well as consuming significant amounts of energy.^18–20^ Due to these drawbacks, alternative and greener extraction methods have been explored based on *e.g.* microwaves^21,22^ and ionic liquids.^23,24^ Unfortunately, these methods often involve the use of strong acids and/or high extraction temperature, which may alter the chemical structure of lignin.

Recently, supercritical fluid technologies have been applied for the extraction of natural compounds from renewable resources such as plants, microalgae, seaweeds and also food by-products.^25–29^ In the field of lignin processing, subcritical and supercritical fluids have been used in depolymerisation reactions^30,31^ but so far not tested in methods for extraction of lignin from lignocellulosic biomass.

Supercritical fluid extraction (SFE) is based on the use of a solvent at temperature and pressure above its critical point. Under this condition the fluid gains a liquid-like density and a gas-like viscosity, which provide fast mass transfer, which often is an advantage in separation processes.^32,33^ CO_2_ is the most commonly used solvent for SFE. The addition of a co-solvent, also known as organic modifier, can be used to increase the relative permittivity of the fluid, which is beneficial for the extraction of polar compounds.^34^ If the organic co-solvent is present in higher amounts in the mixture than the CO_2_ (molar fraction >0.5), the fluid can be defined as a CO_2_-expanded liquid (CXL).^35^ CXLs have a tuneable relative permittivity and the presence of liquid CO_2_ reduces the viscosity of the solvent, thereby enabling faster mass transfer, as compared to the pure co-solvent.^35–37^ Thus, when the extraction mixture contains higher amounts of CO_2_ the extraction is defined as SFE, while with lower amounts of CO_2_ is a CXL extraction (CXLE).

Supercritical and subcritical CO_2_ show attractive physicochemical properties to be used in extraction of lignin. This notwithstanding, SFE and CXL extraction (CXLE) methodology have not been tested for their ability to achieve mild and benign extraction of lignin monomers (LMs) and lignin oligomers (LOs) directly from wood.

In the present study, we investigate the applicability of SFE and CXLE, using different solvent mixtures based on CO_2_, ethanol, acetone, ethyl lactate, and water, for the extraction of lignin from two sources of woodchips.

## Experimental

### Materials

Ethanol (99,7%) was purchased from Solveco (Rosersberg, Sweden), acetone (HPLC grade) from VWR (Radnor, PA, USA), ethyl l-lactate (99%) from Alfa Aesar (Haverhill, MA, USA), methanol (LC-MS grade) from J. T. Baker (Philipsburg, NJ, USA) and 2 M ammonia in methanol from Fisher Scientific (Waltham, MA, USA). Water was purified using a Milli-Q purification system. Liquid CO_2_ (grade 5.3) was obtained from Linde (Dublin, Ireland). Hardwood (oak, *Quercus robur*) and softwood (fir, *Abies alba*) chips were obtained from a local provider in Pisa (Italy). Vanillin, vanillic acid, coniferyl aldehyde, ferulic acid, syringaldehyde, syringic acid, sinapaldehyde, *o*-vanillin, and 3-hydroxyacetophenone were purchased from Sigma Aldrich (St. Louis, MO, USA).

### Sample preparation – calibration solutions

Stock solutions of the LMs were prepared in acetone/water (70/30, v/v) at a concentration of 100 μg mL^−1^ and then diluted to produce calibration solutions in the range of 0.1–50 μg mL^−1^. An internal standard stock solution containing *o*-vanillin, 3-hydroxyacetophenone and 2-hydroxy-3-methoxybenzoic acid at 1 mg mL^−1^ each was used to spike both the LM stock solutions and extracted samples.

### Sample preparation – extraction

**Fig. 1:**
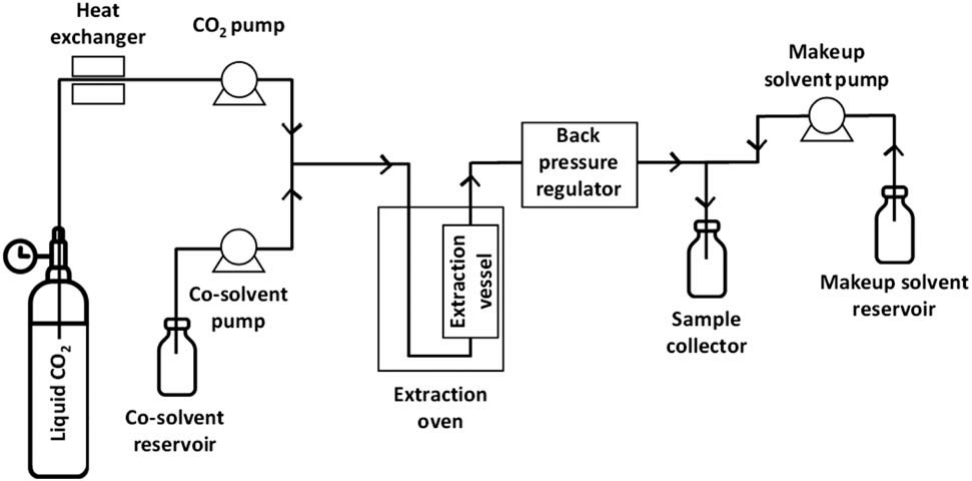
Scheme of the equipment used for CXLE and SFE experiments.

Woodchips were extracted using an analytical SFE system (Waters MV-10, Milford, MA, USA) consisting of a fluid delivery module for pumping CO_2_ and co-solvent, an oven for heating of the extraction vessels, an automated back pressure regulator, a make-up pump, and a fraction collector module, as illustrated in Fig. 1. All the extractions were carried out in dynamic (continuous-flow) mode. For each experiment, 0.501 ± 0.006 g of woodchips were weighted using an analytical balance (four decimals precision). The woodchips, having a particle size of approximately 1 mm, were sandwiched layered in the extraction vessel with glass beads. The heads of the CO_2_ pump were cooled using a chiller operated at 4 °C. The sample was mixed with glass beads and placed into a 10 mL stainless steel extraction vessel. The flow of the two pumps was controlled with the volumetric ratio between CO_2_ and the co-solvent. The pressure and the make-up solvent flow rate were kept at 350 bar and at 0.2 mL min^−1^, respectively. Temperature, CO_2_ percentage and water content in the co-solvent were varied according to an experimental design (see below). After each extraction, the system was flushed for 5 min with a CO_2_/co-solvent mixture that was identical to the one used in the extraction. The collected extracts were evaporated to dryness under a gentle flow of nitrogen gas at room temperature to obtain dry samples that were weighed to determine the gravimetric yield. The solid residue was then re-dissolved in 1.5 mL of acetone/water (70/30, v/v) and then centrifuged at 14 000 rpm for 10 min. The supernatant was collected and stored at −80 °C to minimise possible altering effects in the solvent mixture until analysis. An aliquot of the sample was spiked with the internal standard solution to a final concentration of 100 μg mL^−1^ before UHPSFC/QTOF-MS analysis.

### Hansen solubility parameters (HSP)

Hansen solubility parameters (*δ*), describing the solubility by the similarity between the solute and the solvent, were calculated to provide a theoretical foundation for evaluating lignin solubility. Briefly, similar HSP values of solvent and solute indicate high solubility. For each combination of solvents and lignin, HSP values for ambient conditions were obtained from the literature,^38,39^ or, when unavailable, calculated using the software HSPiP.^40^ The temperature dependence was estimated by the Jayasri and Yaseen equation:^41^
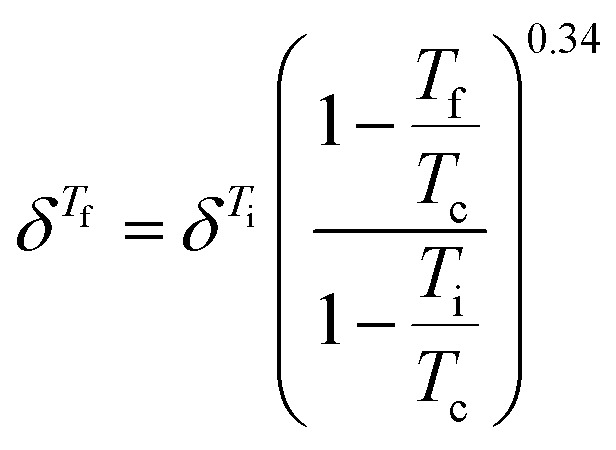
where *T*_c_ is the critical temperature of the solvent, *δ*^*T*_i_^ is the HSP at the reference temperature (*T*_i_) and *δ*^*T*^_f_ is the HSP at the investigated temperature (*T*_f_).

For CO_2_, the effect of the pressure was calculated using the method of Williams *et al.*:^42^
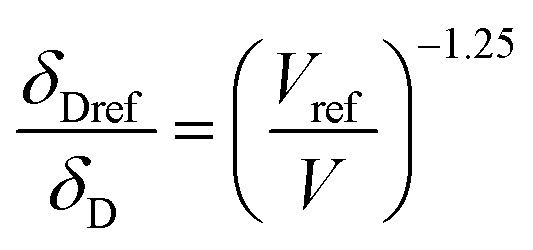

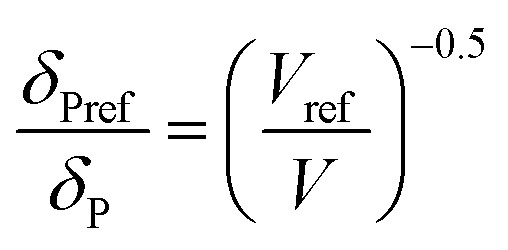

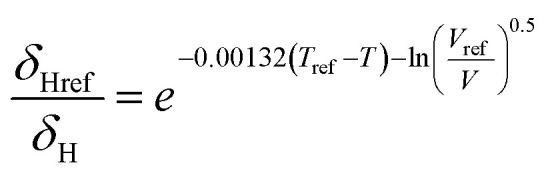
In these equations, the reference parameters are determined using the method of Huang *et al.*^43^ to establish the combination of pressure and molar volume corresponding to the total solubility for pure CO_2_. The molar volume (V) at different pressure and temperature is then calculated by using the molar mass and the density of CO_2_ at the desired pressure and temperature.

For the mixture of solvents, each parameter was calculated as the weighted average of the pure solvent values according to the desired composition of the mixture. The relative energy difference (RED) between lignin and the various solvents was calculated by the following equation:RED = *R*_a_/*R*_0_where *R*_a_ is the difference between the solubility parameters of two substances and *R*_0_ is the maximum solubility parameter difference, which still allows the lignin to be dissolved in the solvent. RED values lower than 1 indicate high affinity.^38^

### Method optimization using design of experiment (DoE)

A full factorial design (FFD) was applied to optimize the influence of the temperature (40–80 °C), the CO_2_ percentage in the extraction mixture (10–90 vol%) and the water content in the co-solvent (0–20 vol%) on the extraction. The FFD was choosen instead of a for example a central composite design (CCD) or a box-Behnken design (BBD) to minimise the total number of required experiments. With three investigated factors and three center points the selected FFD has in total 11 experiments, while a BBD or a CCD with the same number of factors and center points, would have 15 or 17 experiments, respectively. Separate designs were performed for the co-solvents ethanol, acetone and ethyl lactate. The solvent proportions were chosen to ensure that the binary or ternary system generated was in a single liquid phase.^36,44–46^ However, literature data are lacking for the ternary mixture of CO_2_, ethyl lactate and H_2_O.

Partial least squares regression (PLS), using a linear interaction term, was used to evaluate the models with the gravimetric yield (wt%; dry weight of extract/initial sample weight × 100), the number of identified LMs and LDs, and the concentration of vanillin, vanillic acid, coniferyl aldehyde, ferulic acid, syringaldehyde, syringic acid, and sinapaldehyde as responses. The flow rate and the extraction time were kept at 2 mL min^−1^ and 15 min, respectively. In total 11 experiments (Table S1, ESI,†), including three centre point replicates, were performed for each design. Models were evaluated by investigation of the explained variance (R2) and the predictive ability (Q2), obtained by cross-validation. Models showing a Q2 < 0.4 and signs of overfitting (R2 − Q2 > 0.35) were considered unreliable. Models were optimized by removing insignificant terms to yield the highest possible Q2 and lowest degree of overfitting.

The flow rate and the extraction time were optimized. Triplicate experiments were performed at each flow rate investigated (1, 2 and 4 mL min^−1^). During the experiments a fraction of the extracts was collected every 10 min over a period of 50 min. The fractions were dried and the extraction kinetics were visualized by plotting the extracted amount at the five time points (mg of dried extract) *vs.* time and solvent volume.

### Analysis by UHPSFC/QTOF-MS

Quantitative and qualitative analysis of all extracts and standard solutions were performed using a modified method previously reported by our group using ultra-high performance supercritical fluid chromatography coupled with quadrupole-time-of-flight mass spectrometry (UHPSFC/QTOF-MS).^47^ Chromatographic separation was performed with a Waters ultra performance convergence chromatography (UPC^2^) system (Waters, Milford, MA, USA) hyphenated *via* a flow splitter (AQUITY UPC^2^ splitter, Waters) with a Waters XEVO-G2 QTOF-MS (Waters). The injection solvent was acetone/water (70/30 v/v) and 4 μL of samples were injected. The separation was performed with a DIOL column (1.7 μm, 3 mm × 100 mm, Waters) equipped with a torus DIOL VanGuard pre-column (2.1 mm × 5 mm, 1.7 μm, Waters). The separation was achieved using a gradient elution with CO_2_ (A) and methanol (B) as co-solvent with a flow rate of 2 mL min^−1^. The elution gradient started with 0% B (vol%) and then ramped up to 8.5% B (vol%) until 2.5 min, then ramped up to 25% B (vol%) until 5.5 min, then held for 2 min and decreasing to starting composition in 0.5 min. The column temperature was 50 °C and the backpressure was 130 bar. Methanol with 5 mmol per L ammonia was used as a makeup solvent, at a flow rate of 0.5 mL min^−1^. Electrospray ionization (ESI) was performed in negative mode with a capillary voltage of 3.0 kV, a cone voltage of 20 V, a source temperature of 120 °C, a desolvation gas temperature of 600 °C, and a desolvation gas flow rate of 1200 L h^−1^. The mass spectrometer was used in full scan and MS^2^ mode. The *m*/*z* range was *m*/*z* 50–1200. For the MS^2^ measurements a collision-induced dissociation energy ramp from 20 to 35 V was used.

### Evaluation of matrix effects

Possible matrix effects were examined for vanillin using the standard addition method. The sample was extracted in triplicate using the optimised method and then spiked with 0.1, 0.5, 1, 5, and 10 μg mL^−1^ of vanillin, followed by construction of a calibration curve. *o*-Vanillin in a concentration of 1 mg mL^−1^ was used as internal standard. The slope of the standard addition curve was compared with that of the external calibration curve by the Student's *t*-test. An *F*-test was used to ensure homoscedasticity of the data.

### Classification of LMs and LOs

For the classification of LMs and LOs a non-targeted analysis strategy previously developed by our group was used.^48^ The non-targeted analysis strategy is based on the combination of high-resolution mass spectrometry with principal component analysis-quadratic discriminant analysis (PCA-QDA) classification models. First, a feature list including *m*/*z* values was created using the open-source software MZmine 2. Information about the used settings to create the feature list are given in Table S2 (ESI†). Four different PCA-QDA classification models based on literature data for LMs, LDs, trimers (LTRs) and tetramers (LTEs), respectively, were used to determine the number of monomeric units in the features provided in the peak list. The classification models were based on the number of carbon atoms, hydrogen atoms, and oxygen atoms, as well as five Kendrick mass defects with base units of typical functional groups found in lignin-related phenolic compounds. The functional groups were phenol (C_6_H_5_O), methoxy/primary alcohol (CH_3_O), carboxylic acid (CHO_2_), aldehyde (CHO) and secondary alcohol (CH_2_O). If a *m*/*z* value was classified as a LM, LD, LTR or LTE, the ring double bound equivalent (RDB), the mass difference, the detected and theoretical ^13^C/^12^C-intensity ratios and MS^2^ data were used for validation. The result from this classification method is the number of features found for each individual oligomer size (monomers, dimers, *etc.*).

## Results and discussion

### Hansen solubility parameters

HSPs were employed to assess the suitability of several green solvents for lignin extraction. Table S3 (ESI†) reports HSP values for twelve green solvents. In order to obtain a wide range of dispersion, dipole–dipole, and hydrogen bonding interaction strengths, five of these solvents were selected (CO_2_, water, ethanol, ethyl lactate, and acetone) for further investigation. The HSP values calculated for pure solvents and their mixtures were then compared with literature values for lignin.^49^ Then, HSP values for solvents and solvent combinations used in the DoE were correlated with HSP values of some LMs (vanillin, syringaldehyde, vanillic acid, ferulic acid, coniferyl aldehyde, syringic acid and sinapaldehyde). HSP values of the DoE design space for acetone are given in Table 1 and those for ethanol and ethyl lactate in Table S4 (ESI†).

**Table 1:** Hansen solubility parameters, including dispersive interactions (*δ*_D_), polar interactions (*δ*_P_) and hydrogen bonds (*δ*_H_), for lignin, vanillin, pure solvents, and solvent mixtures at the experimental condition in the design space. Values for lignin, vanillin and pure solvents were obtained from the literature.^49^ Values for other LMs were calculated by using the HSPiS software. RED = relative energy difference in relation to HSPs of lignin

Compound(s)	*δ*_D_ (MPa^1/2^)	*δ*_P_ (MPa^1/2^)	*δ*_H_ (MPa^1/2^)	RED	Condition
Lignin	21.9	14.1	16.9		Ambient
Vanillin	19.4	9.8	11.2	0.6	Ambient
Syringaldehyde	19.4	10.5	12.2	0.6	Ambient
Vanillic acid	19.8	8.8	15.4	0.5	Ambient
Ferulic acid	19.3	8.4	15.8	0.6	Ambient
Coniferyl aldehyde	19.3	9.3	11.5	0.6	Ambient
Syringic acid	19.5	8.6	14.9	0.6	Ambient
Sinapaldehyde	19.1	8.9	11.4	0.7	Ambient
Ethanol	15.8	8.8	19.4	1.0	Ambient
Ethyl lactate	16.0	7.6	12.5	1.0	Ambient
Acetone	15.5	10.4	7.0	1.2	Ambient
Water	15.5	16.0	42.3	2.1	Ambient
CO_2_	13.2	4.9	5.4	1.7	25 °C, 350 bar
CO_2_/acetone/H_2_O (50/45/5, v/v/v)	12.9	7.4	7.4	1.6	60 °C, 350 bar
CO_2_/acetone (10/90, v/v)	14.8	9.6	6.7	1.3	40 °C, 350 bar
CO_2_/acetone (10/90, v/v)	13.6	8.9	6.1	1.5	80 °C, 350 bar
CO_2_/acetone (90/10, v/v)	12.7	5.3	5.3	1.7	40 °C, 350 bar
CO_2_/acetone (90/10, v/v)	10.4	4.9	4.7	2.0	80 °C, 350 bar
CO_2_/acetone/H_2_O (10/72/18, v/v/v)	14.9	10.6	12.9	1.1	40 °C, 350 bar
CO_2_/acetone/H_2_O (10/72/18, v/v/v)	13.7	9.9	12.2	1.3	80 °C, 350 bar
CO_2_/acetone/H_2_O (90/8/2, v/v/v)	12.7	5.4	6.0	1.7	40 °C, 350 bar
CO_2_/acetone/H_2_O (90/8/2, v/v/v)	10.4	5.0	5.4	2.0	80 °C, 350 bar

Next, the RED values, estimating the strength of the interaction between lignin and the solvents, were calculated. The design space covers a range from 10.4 to 16.0 for dispersive interactions, 4.9 to 16.0 for polar interactions and 5.2 to 42.3 for hydrogen bond interactions. Lignin and LMs are within the range of polar and hydrogen bond interactions but outside the range of dispersive interactions. The relative energy difference (RED) values between lignin and the LMs and solvents investigated are very similar. For this reason and considering that the extractability of compounds does not only depend on solubility but also on sample matrix effects, all solvents were kept for further investigations.

### Optimisation of the extraction parameters – gravimetric yields

The extraction methods were optimised independently for hardwood (oak) and softwood (fir). Table S5 (ESI†) shows the extraction yield obtained for all performed experiments and Table S6 (ESI†) reports the modelling results for all responses. A two-way analysis of variance (ANOVA) including the two wood types and the three different solvents was performed using the three centre points of each DoE (Table S7†). The results show that there is a significant difference between the gravimetric yields obtained for the two wood types as well as for the different tested solvents. In addition, the predicted values were plotted *vs.* the observed values for each DoE. Linear regression was performed, and the slopes obtained were compared to the slope of 1. The results, reported in Table S9† show that there is no significant difference among the two slopes.

**Fig. 2:**
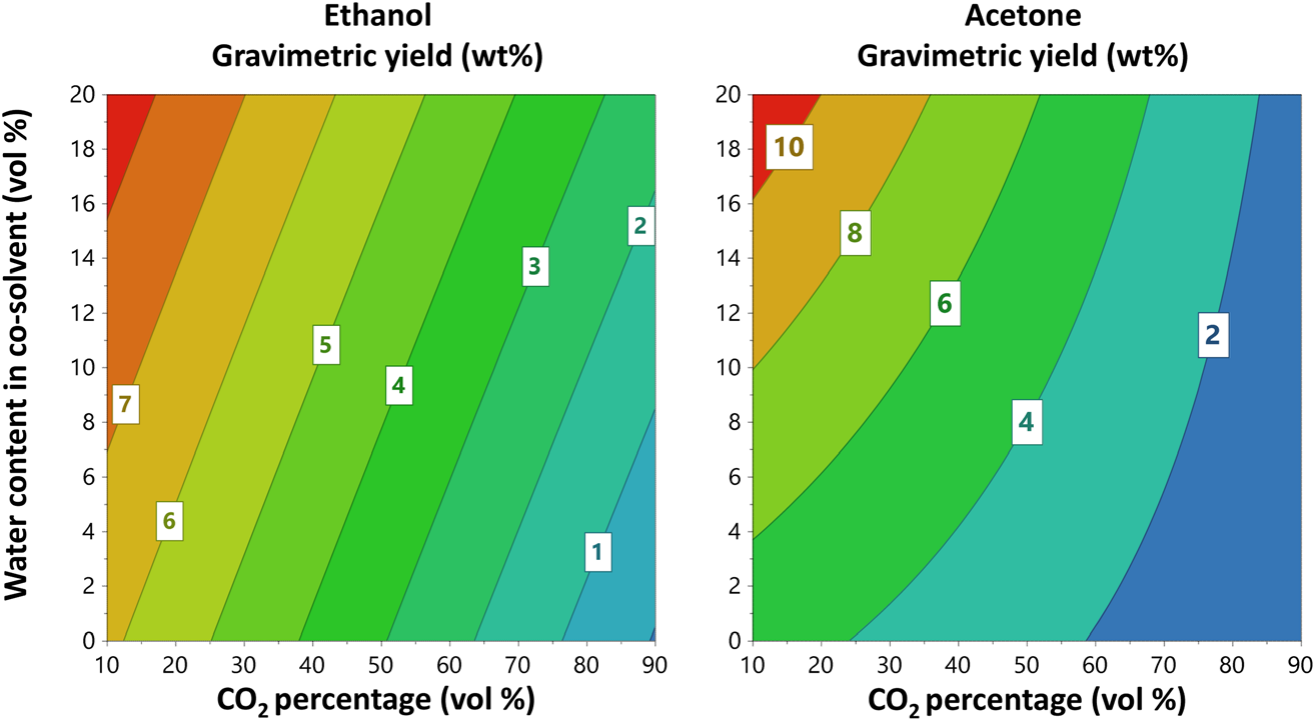
Contour plots from the interaction model obtained using full factorial design showing the influence of the CO_2_ percentage and water content in the co-solvent on the gravimetric yield of oak wood using ethanol and acetone as co-solvent. Ethyl lactate was excluded as provided a non-reliable model. Temperature was 60 °C and pressure 350 bar. Gravimetric yield is expressed as dry weight percent.

**Fig. 3:**
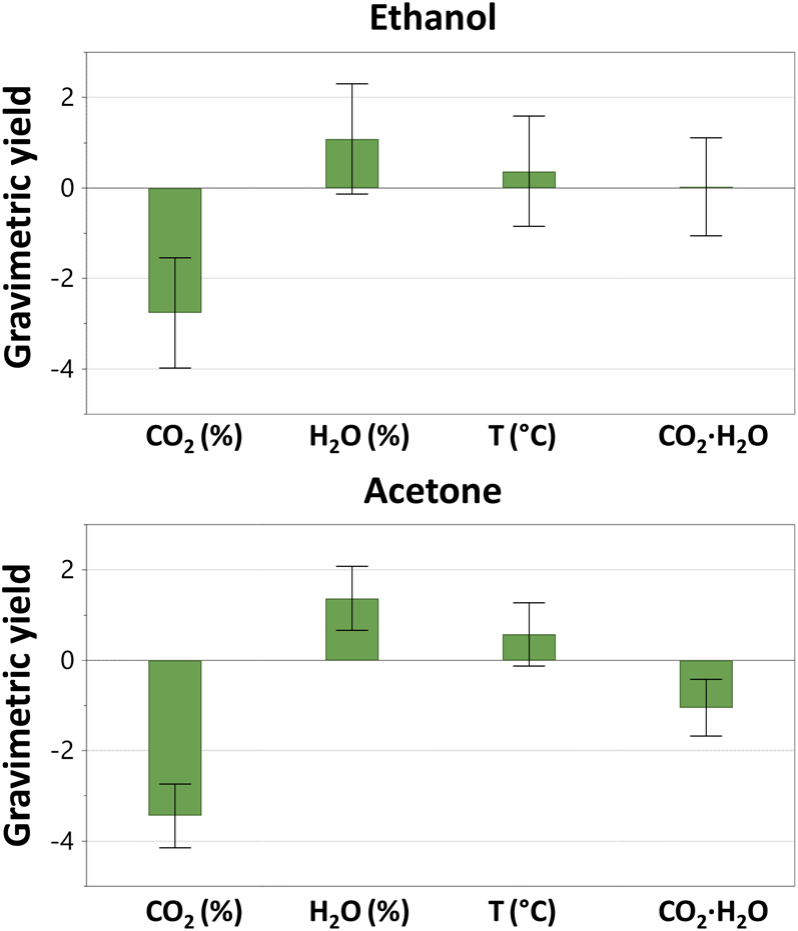
Coefficient plot from the interaction model applied on a full factorial design showing the influence of the investigated factors and two-factor interactions on the gravimetric yield of oak wood using ethanol and acetone as co-solvent. The two-factor interactions with no significant effects for all the solvents were excluded from graphs.

For oak wood, the best gravimetric yields were obtained using ethyl lactate as a co-solvent and the highest yield in the design space was 17.5 weight%. This yield was 4% higher than that obtained for acetone and 7% higher than that for ethanol (*p* = 0.005). Unlikely, in the literature ethanol has been reported as one of the best co-solvents. For instance, Jiang *et al.*^50^ extracted lignin from eucalyptus fibers by using a ternary mixture of scCO_2_, ethanol and water obtaining a yield of 35.9%. However, they used a notably high temperature and extraction time, 180 °C and 60 min, respectively, which could potentially degrade the lignin. The contour plots in Fig. 2, obtained from the oak extraction using each of the respective three co-solvents investigated, show CO_2_ percentage *vs.* water content in the co-solvent at 60 °C. Extraction temperature was, independent of the extraction solvent, without effect on the gravimetric yield for the oak sample (Fig. 2 and 3). As shown in Fig. 2, the presence of water in the co-solvent increases the gravimetric yield in the case of acetone. In addition, lowering the CO_2_ percentage (*i.e.*, increasing the co-solvent amount and thereby also the overall water content of the solvent) has a positive effect on the gravimetric yield from oak wood. This is in line with the effects predicted from analysis of the HSP values (Tables 1 and S4†), *i.e.* when the CO_2_ content is only 10 vol%, the presence of water in the co-solvent yield *δ*_P_ and *δ*_H_ values that are closer to those reported for lignin. Indeed, when the CO_2_ percentage is low, the relative permittivity of the mixture can be adjusted with water allowing the extraction of LMs and LOs. On the contrary, when the CO_2_ is at 90%, the values of *δ*_P_ and *δ*_H_ remain almost the same with or without the presence of water in the co-solvent.

Furthermore, as shown in Fig. 3 there is an interaction effect between the CO_2_ percentage and the water content for acetone. Presumably, this interaction results from hydrogen bonding (see *δ*_H_ in Table 1), which is quite strong with ethanol, followed by ethyl lactate and last acetone. In summary, for all three investigated co-solvents, the CO_2_ percentage appears to be the most influential factor impacting on gravimetric yield, which is likely governed by the water-elicited regulation of the relative permittivity.

The gravimetric yields obtained for softwood (fir) were significantly lower than the yields obtained for hardwood (*p* = 4 × 10^−6^). In this case, only ethyl lactate provided an acceptable model, with models for ethanol and acetone showing very low predictive ability (see Table S6, ESI†). The gravimetric yield was negatively impacted by CO_2_ percentage and water content, while other factors and interactions were insignificant (see Fig. S1 and S2, ESI†).

In summary, the use of ethanol as a co-solvent gives the lowest gravimetric yields for both oak and fir wood, while both acetone and ethyl lactate enable relatively high yields of 5–17.5 weight% from the wood. The CO_2_ percentage should be as low as possible for both wood types, while the water content show wood type-dependent effects. Acetone is the most promising solvent, since evaporation of ethyl lactate, due to its high boiling point (154 °C), requires a time-consuming and energy-demanding evaporation after the extraction, which may also increase the risk of thermal degradation of the target compounds. If evaporation is not needed, then for oak wood, the best extraction solvent in terms of gravimetric yield is CO_2_/ethyl lactate/water (10/72/18, v/v/v) at 60 °C (and 350 bars).

### Characterisation of lignin extracts

Gravimetric analysis provides a rough description of the extraction process, lacking information on compound selectivity and chemical composition of the extracts. Possibly, the presence of high amounts of water may lead to high gravimetric yields due to dissolution of a large proportion of the polysaccharides in the wood, while at the same time reducing the yield of lignin. For this reason, the extracts were analysed using a UHPSFC/ESI-QTOF-MS and a PCA-QDA method to classify, identify and quantify lignin-derived compounds.

**Fig. 4:**
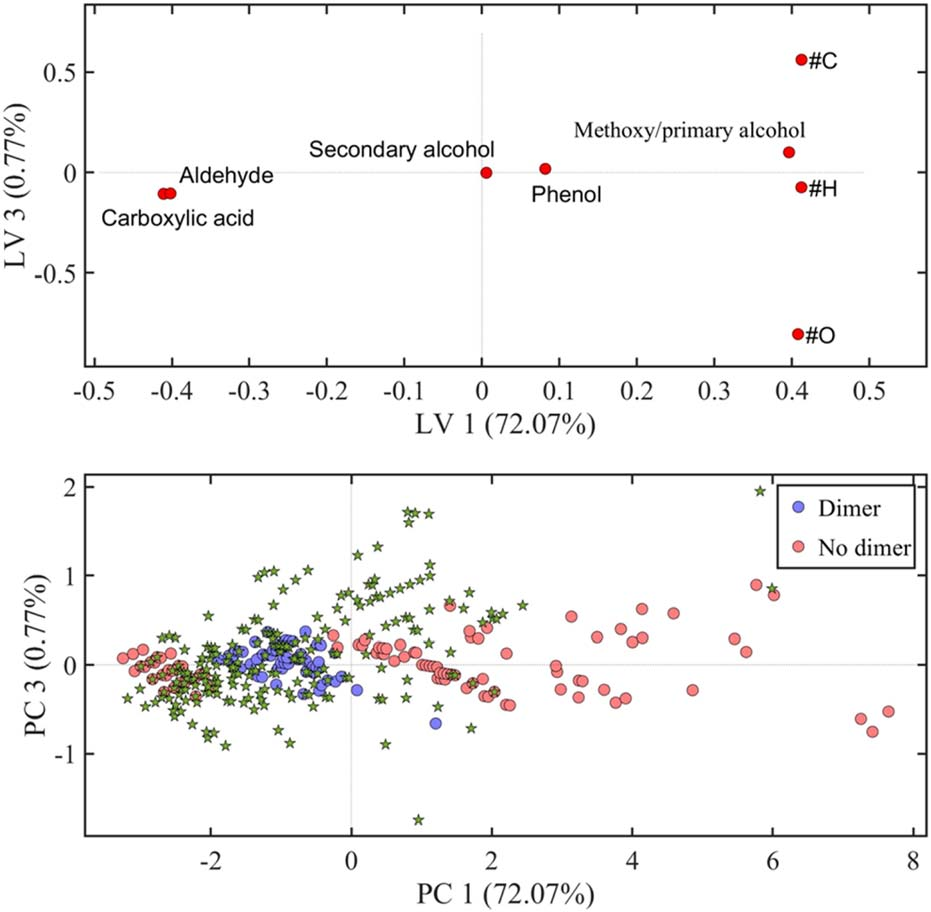
Loading plot (top) and score plot including detected compounds in a centre point measurement of oak wood (bottom) of the KMD-PCA-QDA classification model for lignin compounds showing the latent variables (LVs) 1 and 3. #C = number of carbon atoms, #H = number of hydrogen atoms, #O = number of oxygen atoms. Green stars represent the data obtained from the oak center point as a representative sample.

All the samples turned out to be highly complex, containing more than hundred putative compounds each. Fig. 4 shows the loading plot and score plot for literature data^48^ as well as for one of the oak centre points chosen as a representative sample. The PCA-QDA model provided lists of tentative LMs and LOs, which after further validation were summed up and used as response in the DoE. A two-way ANOVA including the tested solvents and wood types as factors and the number of detected compounds as response was performed using the data obtained by the triplicate measurements form the DoEs center points. As shown in Table S8,† a significant difference of the means is present for both solvents and wood type. Also in this case, the slopes obtained by plotting the predicted values *vs.* the observed values were compared to the slope of 1 showing no significant difference among the two slopes (Table S10†).

Our results show that oak wood extracts contained a significantly higher number of compounds classified as LMs and LOs (*p* = 0.01), with the majority of LOs assigned as LDs, in comparison to fir wood. On average, a similar number of compounds were obtained using acetone and ethyl lactate as co-solvent. Overall, models calculated for the number of identified LMs and LOs showed very low predictability. Only ethyl lactate provided reasonable predictability for both wood types, while acetone provided a reasonable model only for fir.

### Compound-specific extraction yield

To further characterise the extraction selectivity, a number of LMs were quantified and resulting DoE data modelled separately for each LM. For the quantification, the slopes of the external and internal calibration curves for vanillin did not differ as reported in Table S11† (*p* > 0.05 at a 95% confidence), indicating that there are no major matrix effects impacting on its quantification. Consequently, external calibration curves were used throughout this investigation. Linear regression parameters obtained for each calibration curve are summarised in Table S12 (ESI†). The calculated concentrations are reported in Table S13.†

**Fig. 5:**
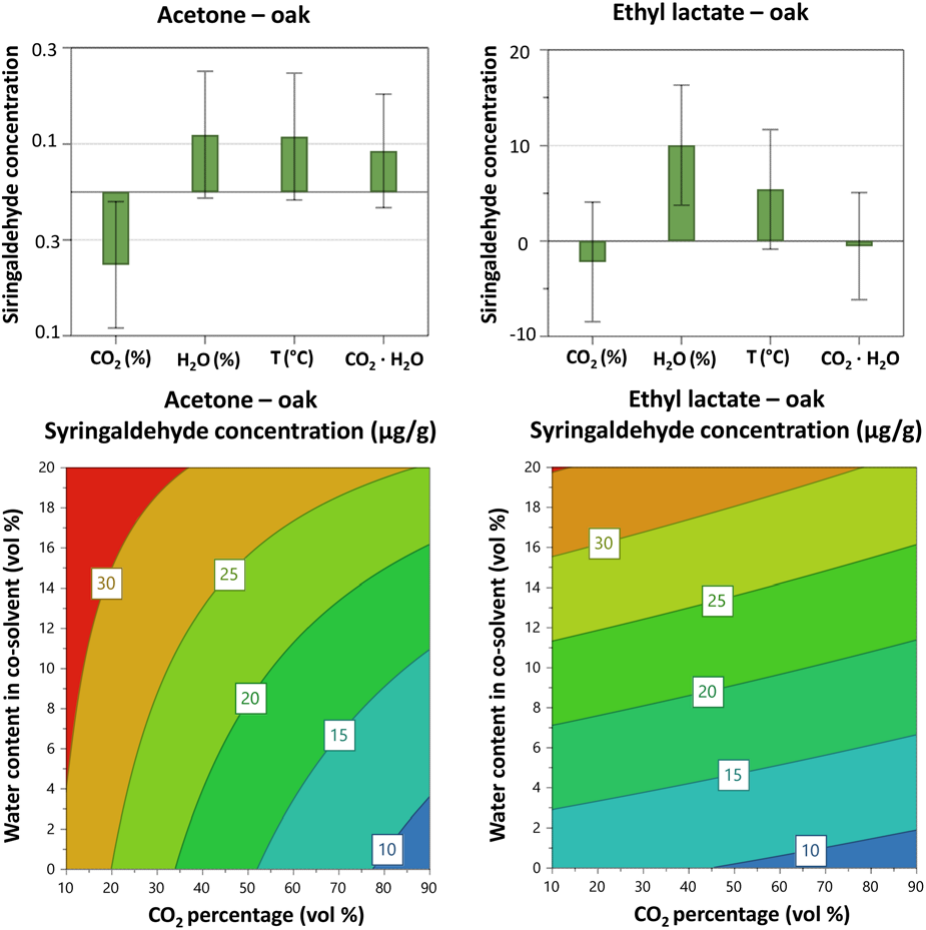
Coefficient plots from the interaction model with a full factorial design showing the influence of the investigated factors and two-factor interactions on the syringaldehyde concentration (μg g^−1^) of oak wood using acetone and ethyl lactate as co-solvent and respective contour plots. CO_2_ (%): CO_2_ percentage; H_2_O (%): water content in co-solvent.

Overall, models showed poor predictability (Table S6, ESI†). Only syringaldehyde and sinapaldehyde provided models with an acceptable predictive ability. For these models, an increased water content in acetone and ethyl lactate was again found be beneficial for increasing the yield of these LMs (Fig. 5 and S9†). The percentage of CO_2_ had a negative influence on the yield of syringaldehyde and sinapaldehyde with acetone and with ethyl lactate as co-solvent. Again, we can compare the values of HSP of the solvent mixtures in the design space with those reported for LMs (Tables 1 and S4†). The use of an extraction mixture with higher amount of water gives values for *δ*_P_ and *δ*_H_ similar to those of LMs, suggesting an enhanced extraction of these analytes in water-rich solvents. On average, ethyl lactate gives slightly higher extraction yields of the LMs than the other co-solvents tested. However, as previously noted, the high boiling point of ethyl lactate still support the use of acetone as a co-solvent.

### Extraction kinetics

**Fig. 6:**
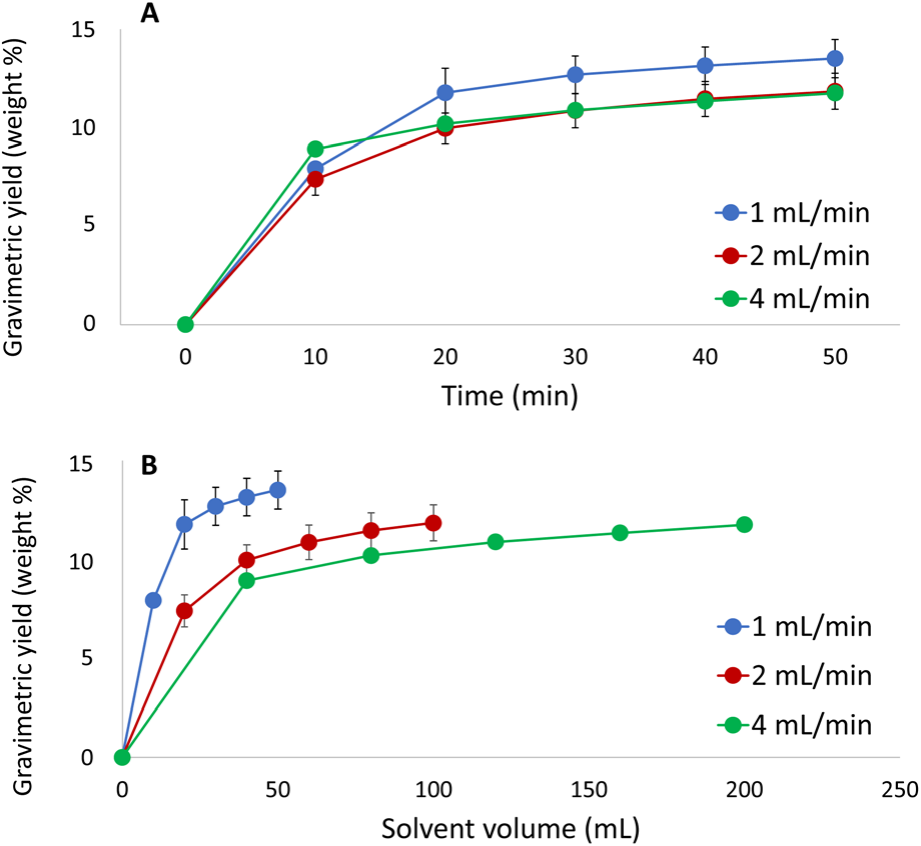
Extracted amount of solute *versus* (A) extraction time and (B) solvent volume at three different flow rates using the optimum extraction conditions.

In order to finalize the optimisation of the method, effects of the extraction time and flow rate on the extraction were investigated using the selected solvent (CO_2_/acetone/water, 10/72/18, v/v/v, at 60 °C and 350 bar (Fig. 6)). As the gravimetric yield was found to reflect the yield of lignin in the preceding investigations, the former was selected as a response due to the convenience of collecting these data. The flow rate was varied to determine which flow rate that provides the fastest extraction rate with a minimum of sample dilution. Moreover, these experiments can be used to determine whether the extraction process is limited by solubility or by mass transfer.

As shown in Fig. 6, complete extraction was achieved within 30 min and the results indicate that the extraction process is controlled by mass transfer rather than solubility. The process is desorption/diffusion-controlled, which implies that running the extraction at higher flow rate will not increase the extracted amount per time unit. However, equilibration and pressurization of the system becomes exceedingly time-consuming when the flow rate is set to the lowest investigated level (1 mL min^−1^). For this reason, extraction at a flow rate of 2 mL min^−1^ for 30 min was chosen as the optimal condition.

### Extraction of lignin from oak wood with the final method

**Fig. 7:**
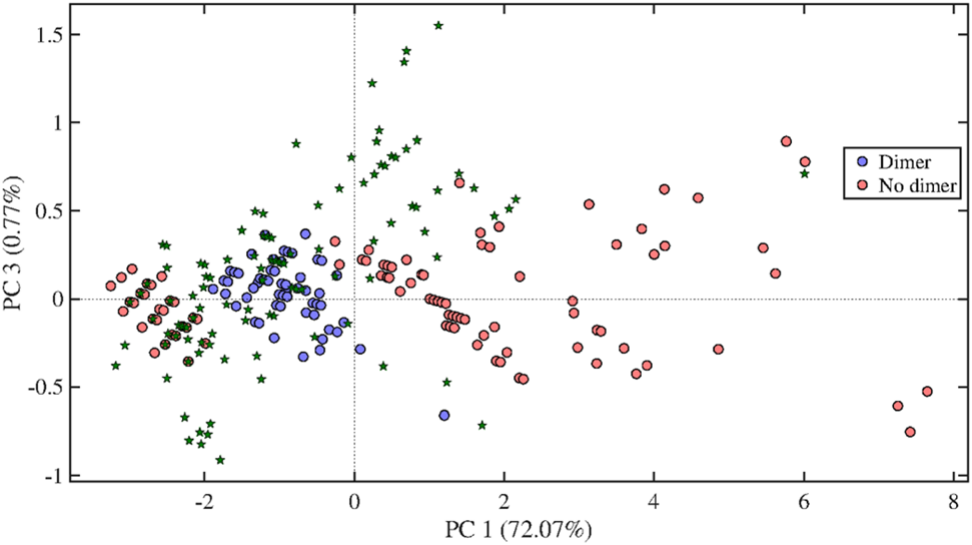
Score plot of the KDM-PCA-QDA classification model for lignin dimers showing the principal components (PCs) 1 and 3. Green stars represent the data obtained from oak wood extraction using the final extraction method.

An oak wood sample was extracted in triplicate with the optimised method having a solvent composition of CO_2_/acetone/water (10/72/18, v/v/v) at 60 °C and 350 bars, for 30 min at 2 mL min^−1^. The extracts were analysed by UHPSFC/ESI-QTOF-MS; the base peak ion chromatogram of one oak sample is shown in Fig. S10.† A total of 104 putative compounds were detected in the resulting data. These compounds were subsequently analysed with the PCA-QDA classification model for LMs and LOs (Fig. 7).

In total, 27 compounds were classified as LMs, 7 as LDs, 9 as LTRs and 7 as LTEs. After validation, 19 compounds were left, out of which 13 were LMs and 6 LDs. The identified LMs and LDs are listed in Table 2. As shown in the table, most of the detected LMs were also identified. However, for three LMs and for all LDs chemical structure could be assigned, which is mainly due to the lack of chemical standards. The concentration of LMs being present at concentrations above the method limit of quantification are presented in Table 2. Among the investigated compounds, vanillin and syringaldehyde are the most easily extractable compounds from oak wood.

**Table 2:** List of LMs and LDs in oak wood classified using the PCA-QDA method. RDB = ring double bond equivalent

Compound	Retention time (min)	*m*/*z* ([M − H]^−^)	Δ*m* (mDa)	Chemical formula	RDB	Concentration^a^ (μg g^−1^)
Vanillin	1.26	151.0389	1.3	C_8_H_8_O_3_	5	8 ± 3
Acetosyringone	1.63	195.0656	1.3	C_10_H_12_O_4_	5	—
Syringaldehyde	1.64	181.0501	0.8	C_9_H_10_O_4_	5	7 ± 2
Coniferyl aldehyde	1.74	177.0552	1.1	C_10_H_10_O_3_	6	2.0 ± 0.2
Cinnamic acid	2.05	147.0449	1.5	C_9_H_8_O_2_	6	—
Sinapaldehyde	2.13	207.0656	1.6	C_11_H_12_O_4_	6	2.0 ± 0.6
Unknown monomer I	2.30	211.0605	0.9	C_10_H_12_O_5_	5	—
*p*-Hydroxybenzaldehyde	2.51	121.0294	1.7	C_7_H_6_O_2_	5	—
Unknown monomer II	3.09	191.0344	1.3	C_10_H_8_O_4_	7	—
Unknown monomer III	3.20	227.0705	0.9	C_14_H_12_O_3_	9	—
Vanillic acid	3.29	167.0346	0.9	C_8_H_8_O_4_	5	—
Syringic acid	3.49	197.0449	0.6	C_9_H_10_O_5_	5	4.30 ± 0.09
Ferulic acid	3.59	193.0500	0.7	C_10_H_10_O_4_	6	1.00 ± 0.09
Unknown dimer I	4.55	317.1024	0.6	C_17_H_18_O_6_	9	—
Unknown dimer II	4.90	385.0920	0.9	C_20_H_18_O_8_	12	—
Unknown dimer III	5.02	359.1488	1.2	C_20_H_24_O_6_	9	—
Unknown dimer IV	5.08	319.1175	1.2	C_17_H_20_O_6_	8	—
Unknown dimer V	5.40	419.1708	0.3	C_22_H_28_O_8_	9	—
Unknown dimer VI	6.33	303.0510	0.0	C_15_H_12_O_7_	10	—

a Concentrations based on external calibration and expressed in dry weight of sample.

## Conclusions

In the presented work, a new SFE- and CXLE-based method for extraction of lignin from wood chips has been thoroughly developed and optimized. The method allowed the extraction of LMs and LDs from hardwood and softwood chips. Either acetone and ethyl lactate are good co-solvents within the investigated parameter ranges. Furthermore, the addition of water to the co-solvent is beneficial for the extraction of lignin, while the extraction temperature showed no positive significant influence.

Future perspective of the work will be the extraction of lignin from various lignocellulosic matrices such as grass and different species of hardwood and softwood. In addition, the use of this method to study biomass subjected to pre-treatments would provide interesting information on the compositional modification of the extracted lignin. Moreover, a deeper investigation of the selectivity of the tested co-solvents would potentially allow us to achieve more accurate quantitative results both for gravimetric yield and LM concentration.

## Author contributions

FN performed experimental work and wrote the first draft of the manuscript. JP contributed to the experimental work and drafting of the manuscript. ER, MS, PS and CT critically discussed results of the study. All authors contributed to writing the final version of the manuscript.

## Conflicts of interest

There are no conflicts to declare.

## Supplementary Material

RA-013-D3RA01873C-s001
