# Supplementary material for: Native lignin extraction from soft- and hardwood by green and benign sub/supercritical fluid extraction methodologies
Source: RSC Adv. 2023 Jul 20;13(32):21945–53. doi: 10.1039/d3ra01873c (PMC10357412; doi:10.1039/d3ra01873c)

Electronic Supplementary Material (ESI) for RSC Advances.
This journal is © The Royal Society of Chemistry 2023

Native lignin extraction from soft- and hardwood by green and benign sub/supercritical fluid extraction methodologies –Supplementary Information (SI)

Federica Nardella [a]†, Jens Prothmann [b]† , Margareta Sandahl [b], Peter, Spégel[b], Erika Ribechini [a], and Charlotta Turner *[b]

[1] Department of Chemistry and Industrial Chemistry, University of Pisa, Via G. Moruzzi 13, 56124, Pisa, Italy

[2] Lund University, Department of Chemistry, Centre for Analysis and Synthesis, Lund, Sweden

† Both authors contributed equally

* Corresponding author. Charlotta.Turner@chem.lu.se

Content

Table S1. Values for the studied variables set (temperature, CO_2 ratio and water content in the co-solvent) for the 11 experiments performed as part of the DOE. The run order was randomised. 3

Table S2. Settings to create the feature list in MZmine 2. 4

Table S3. Hansen solubility parameters, including dispersive interactions (δD), polar interactions (δP), hydrogen bonds (δH), and relative energy difference related to lignin (RED) for several green solvents (Hansen, 2007). 5

Table S4. Hansen solubility parameters, including dispersive interactions (δD), polar interactions (δP) and hydrogen bonds (δH), for solvent mixtures at the experimental condition in the DOE design space for ethanol and ethyl lactate. RED =Relative energy difference related to lignin; EtOH = ethanol, ELac = ethyl lactate. 6

Table S5. Gravimetric yields obtained for both oak and fir extracted with the three investigated solvents. The yield was calculated as % dry extract weight obtained/ initial sample weight, w/w. CP = centre point. Run order was randomised. 7

Figure S1. Softwood (fir) coefficient plot and contour plots obtained from the DOE for ethyl lactate, showing the effect of CO_2 ratio and water content in the co-solvent on the gravimetric yield. Model obtained for other solvents were invalid. 8

Table S6. Model fit of the data obtained for all the DOE performed and different responses. R^2 is the percent of the variation of the response explained by the model. Q^2 is the percent of the variation of the response predicted by the model according to cross validation. 9

Table S7. ANOVA results comparing the gravimetric yields obtained by the center points of each DoE, which were performed as triplicates. 11

Table S8. ANOVA results comparing the number of detected compounds obtained by the center points of each DoE, which were performed as triplicates. 12

Table S9. Results from comparison of the slopes obtained by plotting predicted vs observed values with the slope of 1 for each DoE of gravimetric yield. 13

Table S10. Results from comparison of the slopes obtained by plotting predicted vs observed values with the slope of 1 for each DoE of number of identified compounds. 14

Table S11. Comparison and statistics of standard addition and external calibration curve. 15

Table S12. Calibration curves obtained by linear regression using standards of LMs. 15

Table S13. Concentration of vanillin, vanillic acid, coniferyl aldehyde, ferulic acid, syringaldehyde, syringic acid and sinapaldehyde obtained in the investigated extracts. Being a softwood, fir wood does not contain

syringaldehyde, syringic acid and sinapaldehyde. (<LOD): the concentration was below the LOD of method. 16

Figure S2. Coefficient plots and respective contour plots showing the influence of the investigated factors on the sinapaldehyde concentration (µg/g) of oak and fir wood. 18

Figure S3. Base peak ion chromatogram of the lignin extracted from oak wood obtained with UHPSFC/ESI-HRMS. 19

Figure S4. Loading plot of the KMD-PCA-QDA classification model for lignin dimers showing the latent variables (LVs) 1 and 3. #C = number of carbon atoms, #H = number of hydrogen atoms, #O = number of oxygen atoms. 20

Table S1. Values for the studied variables set (temperature, CO_2 ratio and water content in the co-solvent) for the 11 experiments performed as part of the DOE. The run order was randomised.

	T (°C)	CO_2 ratio (vol%)	Water content in co-solvent (vol%)
**Centre point (n=3)**	60	50	10
**Experiment 1**	40	10	0
**Experiment 2**	80	10	0
**Experiment 3**	40	90	0
**Experiment 4**	80	90	0
**Experiment 5**	40	10	20
**Experiment 6**	80	10	20
**Experiment 7**	40	90	20
**Experiment 8**	80	90	20

Table S2. Settings to create the feature list in MZmine 2.

1. Raw data methods/Peak detection
    - 1.1 Mass detection
        - Settings:
            - Polarity: negative
            - Mass detector: centroid
            - Spectrum type: any
            - Noise level: 1.0E4
    - 1.2 Chromatogram builder
        - Settings:
            - MS level: 1
            - Polarity: negative
            - Spectrum type: any
            - Min time span: 0.03 min
            - Min height: 3.0E4
            - m/z tolerance: 0.01 Da or 5.0 ppm
2. Peak list methods
    - 2.1 Peak detection/chromatogram deconvolution
        - Settings:
            - Algorithm: local minimum search
            - Chromatographic threshold: 30.0%
            - Minimum relative height: 1.0%
            - Minimum absolute height: 1.0E4
            - Min ratio of peak top/edge 2
            - m/z center calculation: median
    - 2.2 Isotopes/isotopes peak grouper
        - Settings:
            - m/z tolerance: 0.01 Da or 10.0 ppm
            - Retention time tolerance: 0.05 min
            - Maximum charge: 1
            - Representative isotope: most intense

    - 2.3 Identification/adduct search
        - Settings:
            - Retention time tolerance: 0.05 min
            - Adducts: [M+NH3], 17.0265 Da; [M+H3PO4], 97.9769 Da; [M+H2SO4], 97.9674; [M+CH2O2], 46.0056
            - m/z tolerance: 0.01 Da or 5 ppm
            - Max relative adduct peak height: 50.0%
    - 2.4 Identification/formula prediction
        - Settings:
            - Charge: 1
            - Ionisation type: [M-H]-
            - m/z tolerance: 0.005 Da or 5.0 ppm
            - Max best formulas per peak: 1
            - Elements: C, O, H, S (for all min: 0, max: 100)
            - Element count heuristics: H/C ratio: yes, multiple element counts: yes
            - RDBE restrictions: RDBE range: -1 to 40, RDBE must be an integer: yes

Table S3. Hansen solubility parameters, including dispersive interactions (δD), polar interactions (δP), hydrogen bonds (δH), and relative energy difference related to lignin (RED) for several green solvents (Hansen, 2007).

	δD **(MPa$^{1/2}$)**	***δP*** **(MPa$^{1/2}$)**	***δH*** **(MPa$^{1/2}$)**	**RED**	**Condition**
CO_2	13.2	4.9	5.4	1.7	25 °C, 350 bar
Water	15.5	16.0	42.3	2.1	Ambient
Methanol	15.1	12.3	22.3	1.1	Ambient
Ethanol	15.8	8.8	19.4	1.0	Ambient
1-butanol	16.0	5.7	15.8	1.1	Ambient
Ethyl acetate	15.8	5.3	7.2	1.3	Ambient
Isopropyl acetate	14.9	4.5	8.2	1.4	Ambient
Ethyl lactate	16.0	7.6	12.5	1.0	Ambient
Acetone	15.5	10.4	7.0	1.2	Ambient
Ethylene glycol	17.0	11.0	26	1.0	Ambient
Anisole	17.8	4.1	6.7	1.2	Ambient
MEK (methyl ethyl ketone)	16.0	9.0	5.1	1.3	Ambient

Table S4. Hansen solubility parameters, including dispersive interactions (δD), polar interactions (δP) and hydrogen bonds (δH), for solvent mixtures at the experimental condition in the DOE design space for ethanol and ethyl lactate. RED =Relative energy difference related to lignin; EtOH = ethanol, ELac = ethyl lactate.

	δD **($MPa^{1/2}$)**	***δP*** **($MPa^{1/2}$)**	***δH*** **($MPa^{1/2}$)**	**RED**	**Condition**
CO_2/EtOH/H_2O (50/45/5, v/v/v)	13.0	6.8	12.7	1.4	60 °C, 350 bar
CO_2/EtOH (10/90, v/v)	15.1	8.2	17.6	1.1	40 °C, 350 bar
CO_2/EtOH (10/90, v/v)	13.9	7.6	16.2	1.3	80 °C, 350 bar
CO_2/EtOH (90/10, v/v)	12.7	5.1	6.6	1.7	40 °C, 350 bar
CO_2/EtOH (90/10, v/v)	10.4	4.7	5.8	2.0	80 °C, 350 bar
CO_2/EtOH/ H_2O (10/72/18, v/v/v)	15.1	9.5	21.6	1.1	40 °C, 350 bar
CO_2/EtOH/ H_2O (10/72/18, v/v/v)	13.9	8.9	20.3	1.2	80 °C, 350 bar
CO_2/EtOH/ H_2O (90/8/2, v/v/v)	12.7	5.3	7.0	1.7	40 °C, 350 bar
CO_2/EtOH/ H_2O (90/8/2, v/v/v)	10.4	4.9	6.3	2.0	80 °C, 350 bar
CO_2/EtLac/H_2O (50/45/5, v/v/v)	13.2	6.3	9.9	1.5	60 °C, 350 bar
CO_2/EtLac (10/90, v/v)	15.4	7.2	11.6	1.1	40 °C, 350 bar
CO_2/EtLac (10/90, v/v)	14.4	6.8	10.9	1.3	80 °C, 350 bar
CO_2/EtLac (90/10, v/v)	12.7	5.0	5.9	1.7	40 °C, 350 bar
CO_2/EtLac (90/10, v/v)	10.5	4.6	5.2	2.0	80 °C, 350 bar
CO_2/EtLac/ H_2O (10/72/18, v/v/v)	15.3	8.7	16.9	1.0	40 °C, 350 bar
CO_2/EtLac/ H_2O (10/72/18, v/v/v)	14.4	8.2	16.0	1.2	80 °C, 350 bar
CO_2/EtLac/ H_2O (90/8/2, v/v/v)	12.7	5.2	6.5	1.7	40 °C, 350 bar
CO_2/EtLac/ H_2O (90/8/2, v/v/v)	10.5	4.8	5.8	2.0	80 °C, 350 bar

Table S5. Gravimetric yields obtained for both oak and fir extracted with the three investigated solvents. The yield was calculated as % dry extract weight obtained/ initial sample weight, w/w. CP = centre point. Run order was randomised.

	Ethanol		Acetone		Ethyl lactate	
	Oak (%)	Fir (%)	Oak (%)	Fir (%)	Oak (%)	Fir (%)
**CP (average)**	3.1	0.9	4.3	1.3	5.3	2.2
**Experiment 1**	5.5	0.9	5.2	1.0	9.1	5.3
**Experiment 2**	7.8	1.1	5.6	0.9	8.5	6.0
**Experiment 3**	0.3	0.7	0.3	0.7	7.4	3.0
**Experiment 4**	0.5	0.8	0.4	0.7	0.4	3.1
**Experiment 5**	8.0	1.1	9.1	11.0	11.3	1.9
**Experiment 6**	10.0	2.6	13.1	1.7	17.5	3.7
**Experiment 7**	3.5	0.8	0.4	0.9	0.3	1.6
**Experiment 8**	2.3	0.9	1.1	0.7	3.5	1.7

Figure S1. Softwood (fir) coefficient plot and contour plots obtained from the DOE for ethyl lactate, showing the effect of CO_2 ratio and water content in the co-solvent on the gravimetric yield. Model obtained for other solvents were invalid.

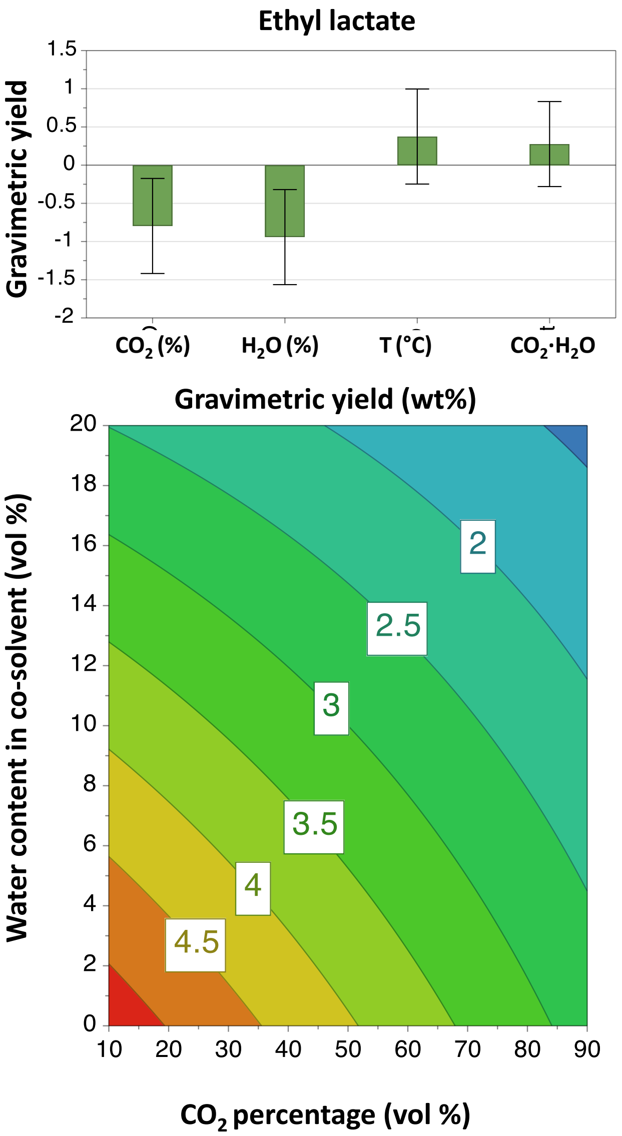

Figure S1 - Coefficient plots from the interaction model with a full factorial design showing the influence of the investigated factors and two-factor interactions on the gravimetric yield and contour plots showing the influence of the CO_2 percentage and water content in the co-solvent on the gravimetric yield of fir wood using ethyl lactate as co-solvent. CO_2 (%): CO_2 percentage; H_2O (%): water content in co-solvent. Temperature was 60 °C and pressure 350 bar.

Table S6. Model fit of the data obtained for all the DOE performed and different responses. R^2 is the percent of the variation of the response explained by the model. Q^2 is the percent of the variation of the response predicted by the model according to cross validation.

	Gravimetric yield (wt%)					
	Ethanol		Acetone		Ethyl lactate	
	Oak	Fir	Oak	Fir	Oak	Fir
R^2	0.86	0.64	0.97	0.63	0.91	0.82
Q^2	0.60	0.23	0.73	0.07	0.48	0.58
R^2-Q^2	0.26	0.41	0.24	0.56	0.43	0.24
Reproducibility	0.78	0.54	1.00	1.00	0.97	0.94
	**Number of identified compounds**					
	Ethanol		Acetone		Ethyl lactate	
	Oak	Fir	Oak	Fir	Oak	Fir
R^2	0.46	0.55	0.22	0.20	0.66	0.64
Q^2	-0.19	0.30	-0.13	-0.20	0.39	0.39
R^2-Q^2	0.66	0.24	0.35	0.40	0.26	0.26
Reproducibility	0.43	-0.20	0.60	0.42	0.97	0.08
	**Vanillin concentration (µg/g)**					
	Ethanol		Acetone		Ethyl lactate	
	Oak	Fir	Oak	Fir	Oak	Fir
R^2	0.50	0.85	0.33	0.24	0.94	0.70
Q^2	-0.20	0.18	-0.11	-0.20	0.46	0.01
R^2-Q^2	0.70	0.68	0.44	0.44	0.49	0.69
Reproducibility	0.97	0.83	-0.20	0.95	0.94	0.53
	**Vanillic acid concentration (µg/g)**					
	Ethanol		Acetone		Ethyl lactate	
	Oak	Fir	Oak	Fir	Oak	Fir
R^2	0.59	0.69	0.42	0.67	0.87	0.72
Q^2	-0.01	0.17	0.06	-0.07	0.28	0.13
R^2-Q^2	0.60	0.52	0.35	0.74	0.59	0.59
Reproducibility	0.88	0.68	-0.20	0.89	0.73	0.92
	**Coniferyl aldehyde concentration (µg/g)**					
	Ethanol		Acetone		Ethyl lactate	
	Oak	Fir	Oak	Fir	Oak	Fir
R^2	0.74	0.46	0.65	0.23	0.91	0.86
Q^2	0.20	0.26	0.10	-0.20	0.41	0.37
R^2-Q^2	0.54	0.20	0.56	0.43	0.50	0.49
Reproducibility	0.39	-0.20	0.83	0.92	0.55	0.37
	**Ferulic acid concentration (µg/g)**					
	Ethanol		Acetone		Ethyl lactate	
	Oak	Fir	Oak	Fir	Oak	Fir
R^2	0.36	0.35	0.20	0.93	0.52	0.81
Q^2	0.30	-0.16	-0.20	0.28	-0.03	0.27
R^2-Q^2	0.07	0.51	0.40	0.65	0.55	0.53
Reproducibility	-0.20	-0.20	1.00	1.00	0.17	0.80

	Syringaldehyde concentration (μg/g)		
	**Ethanol**	**Acetone**	**Ethyl lactate**
	**Oak**	**Oak**	**Oak**
**R^2**	0.34	0.77	0.77
**Q^2**	-0.20	0.57	0.45
**R^2-Q^2**	0.54	0.20	0.32
**Reproducibility**	-0.14	0.90	1.00
	**Syringic acid concentration (μg/g)**		
	**Ethanol**	**Acetone**	**Ethyl lactate**
	**Oak**	**Oak**	**Oak**
**R^2**	0.64	0.28	0.09
**Q^2**	0.20	-0.19	-0.20
**R^2-Q^2**	0.44	0.47	0.29
**Reproducibility**	0.74	0.09	-0.20
	**Sinapaldehyde concentration (μg/g)**		
	**Ethanol**	**Acetone**	**Ethyl lactate**
	**Oak**	**Oak**	**Oak**
**R^2**	0.01	0.77	0.73
**Q^2**	-0.14	0.44	0.33
**R^2-Q^2**	0.15	0.32	0.40
**Reproducibility**	-0.20	0.81	0.81

Table S7. ANOVA results comparing the gravimetric yields obtained by the center points of each DoE, which were performed as triplicates.

Gravimetric yield (wt%)	Ethanol	Acetone	Ethyl lactate
**Oak**	1.4	4.48	4.35
	3.4	4.29	5.89
	4.4	4.16	5.62
**Fir**	0.77	1.31	2.48
	0.55	1.21	1.8
	1.28	1.31	2.34

SUMMARY	Ethanol	Acetone	Ethyl lactate	Total
Oak				
**Count**	3	3	3	9
**Sum**	9.2	12.93	15.86	37.99
**Average**	3.066667	4.31	5.286667	4.221111
**Variance**	2.333333	0.0259	0.676233	1.687386
Fir				
**Count**	3	3	3	9
**Sum**	2.6	3.83	6.62	13.05
**Average**	0.866667	1.276667	2.206667	1.45
**Variance**	0.140233	0.003333	0.128933	0.4217
Total				
**Count**	6	6	6	
**Sum**	11.8	16.76	22.48	
**Average**	1.966667	2.793333	3.746667	
**Variance**	2.441427	2.772027	3.167987	

ANOVA

Source of Variation	*SS*	*df*	*MS*	*F*	*P-value*	*F crit*
**Wood types**	34.55576	1	34.55576	62.67733	4.183E-06	4.7472253
**Solvents**	9.521244	2	4.760622	8.634831	0.0047488	3.8852938
**Interaction**	0.735511	2	0.367756	0.667036	0.5312643	3.8852938
**Within**	6.615933	12	0.551328			
**Total**	51.42844	17				

Table S8. ANOVA results comparing the number of detected compounds obtained by the center points of each DoE, which were performed as triplicates.

Number of identified compounds	Ethanol	Acetone	Ethyl lactate
**Oak**	17	24	18
	18	39	20
	11	34	20
**Fir**	20	13	15
	5	24	7
	9	20	18

SUMMARY	Ethanol	Acetone	Ethyl lactate	Total
Oak				
**Count**	3	3	3	9
**Sum**	46	97	58	201
**Average**	15.33333	32.33333	19.33333	22.33333
**Variance**	14.33333	58.33333	1.333333	77.75
Fir				
**Count**	3	3	3	9
**Sum**	34	57	40	131
**Average**	11.33333	19	13.33333	14.55556
**Variance**	60.33333	31	32.33333	42.77778
Total				
**Count**	6	6	6	
**Sum**	80	154	98	
**Average**	13.33333	25.66667	16.33333	
**Variance**	34.66667	89.06667	24.26667	

ANOVA

Source of Variation	*SS*	*df*	*MS*	*F*	*P-value*	*F crit*
**Wood types**	272.2222	1	272.2222	8.263069	0.0139711	4.7472253
**Solvents**	496.4444	2	248.2222	7.53457	0.00759	3.8852938
**Interaction**	72.44444	2	36.22222	1.099494	0.3643704	3.8852938
**Within**	395.3333	12	32.94444			
**Total**	1236.444	17				

Table S9. Results from comparison of the slopes obtained by plotting predicted vs observed values with the slope of 1 for each DoE of gravimetric yield.

	Gravimetric yield											
	Ethanol				Acetone				Ethyl lactate			
	Oak		Fir		Oak		Fir		Oak		Fir	
	Pred	Obs	Pred	Obs	Pred	Obs	Pred	Obs	Pred	Obs	Pred	Obs
**Exp 1**	-0.47	0.30	0.44	0.73	-0.38	0.27	-1.00	0.94	1.13	0.26	0.98	1.64
**Exp 2**	0.36	0.50	0.58	0.83	0.54	0.41	-0.91	0.74	1.60	3.54	1.82	1.75
**Exp 3**	2.03	3.50	0.72	0.89	0.77	0.39	-0.41	0.65	3.09	7.37	2.07	1.90
**Exp 4**	2.86	2.30	0.91	0.83	1.69	1.14	1.92	0.96	3.56	0.35	2.40	2.99
**Exp 5**	4.28	1.40	1.05	0.90	4.38	4.48	1.95	1.31	6.71	4.35	2.91	3.72
**Exp 6**	4.28	3.40	1.06	0.77	4.38	4.29	1.95	1.21	6.71	5.89	3.00	2.48
**Exp 7**	4.28	4.40	1.06	0.55	4.38	4.16	1.95	1.31	6.71	5.62	3.00	1.80
**Exp 8**	5.78	5.50	1.06	1.28	4.39	5.23	2.01	0.74	8.05	9.11	3.00	2.34
**CP1**	6.61	7.80	1.20	1.13	5.53	5.58	2.51	0.92	8.52	8.53	3.24	3.11
**CP2**	8.13	8.00	1.55	1.13	10.66	9.11	4.28	1.66	13.61	11.27	4.87	5.27
**CP3**	8.96	10.00	2.03	2.61	11.81	13.08	7.20	11.00	14.08	17.48	5.71	6.00
**Slope**	1.000000		0.999999		1.000002		1.000000		0.999997		1.000000	
**n**	11		11		11		11		11		11	
**SE (reg)**	1.28155138		0.353123537		0.794891708		1.98170723		2.553638349		0.656485923	
**SE(slope)**	0.135543709		0.252758419		0.064121878		0.265557111		0.186672217		0.15660464	
**Difference**	1.1E-07		1.4E-06		2.3E-06		2.4E-07		2.8E-06		3.2E-07	
**SE (diff)**	0.135543709		0.252758419		0.064121878		0.265557111		0.186672217		0.15660464	
**t-stat**	7.9E-07		5.6E-06		3.5E-05		9.1E-07		1.5E-05		2.0E-06	
**df**	14		14		14		14		14		14	
**p**	0.99999938		0.999995597		0.999972439		0.999999284		0.999988147		0.999998423	

Table S10. Results from comparison of the slopes obtained by plotting predicted vs observed values with the slope of 1 for each DoE of number of identified compounds.

	**Number of identified compounds**											
	**Ethanol**				**Acetone**				**Ethyl lactate**			
	**Oak**		**Fir**		**Oak**		**Fir**		**Oak**		**Fir**	
	**Pred**	**Obs**	**Pred**	**Obs**	**Pred**	**Obs**	**Pred**	**Obs**	**Pred**	**Obs**	**Pred**	**Obs**
**Exp 1**	13.70	8.00	6.37	5.00	14.42	8.00	7.91	2.00	10.27	9.00	5.43	4.00
**Exp 2**	9.20	8.00	5.25	8.00	8.52	2.00	4.99	2.00	4.77	3.00	4.83	3.00
**Exp 3**	19.20	21.00	13.42	12.00	23.67	17.00	14.26	13.00	21.27	18.00	17.40	19.00
**Exp 4**	17.20	21.00	7.53	8.00	10.42	7.00	12.33	8.00	10.77	8.00	9.31	11.00
**Exp 5**	12.95	18.00	13.72	17.00	19.36	13.00	7.80	7.00	13.27	10.00	7.31	8.00
**Exp 6**	8.45	9.00	12.60	9.00	13.46	8.00	4.88	1.00	7.77	5.00	6.71	4.00
**Exp 7**	18.45	16.00	20.77	22.00	28.61	24.00	14.15	9.00	24.27	23.00	19.28	16.00
**Exp 8**	16.45	12.00	14.88	15.00	15.36	8.00	12.22	9.00	13.77	12.00	11.19	7.00
**CP1**	14.45	17.00	11.82	20.00	16.73	24.00	9.82	13.00	13.27	18.00	10.18	15.00
**CP2**	14.45	18.00	11.82	5.00	16.73	39.00	9.82	24.00	13.27	20.00	10.18	7.00
**CP3**	14.45	11.00	11.82	9.00	16.73	34.00	9.82	20.00	13.27	20.00	10.18	18.00
**Slope**	1.000005		1.000002		1.000005		1.000000		1.000001		1.000000	
**n**	11		11		11		11		11		11	
**SE (reg)**	3.86188		4.19377		11.21952		6.92611		4.20558		3.96305	
**SE(slope)**	0.3578		0.3064		0.6259		0.6730		0.2416		0.2746	
**Difference**	4.55E-06		2.17E-06		5.16E-06		5.00E-07		9.36E-07		2.48E-07	
**SE (diff)**	0.3578		0.3064		0.6259		0.6730		0.2416		0.2746	
**t-stat**	1.27E-05		7.09E-06		8.25E-06		7.42E-07		3.87E-06		9.03E-07	
**df**	14		14		14		14		14		14	
**p**	0.999990		0.999994		0.999994		0.999999		0.999997		0.999999	

Table S11. Comparison and statistics of standard addition and external calibration curve.

	Slope	Slope error	Intercept	Intercept error	t calculated	t listed (α=0.05)
**External**	0.10827	0.00154	-0.00629	0.00825	1.04	2.31
**Standard addition**	0.09927	0.01937	0.43518	0.11545		

Table S12. Calibration curves obtained by linear regression using standards of LMs.

Compound	Slope	Slope error	Intercept	Intercept error	R^2
Vanillin	0.108	0.00154	-0.006	0.00825	0.999
Vanillic acid	0.001	0.00003	0.001	0.00062	0.998
Coniferyl aldehyde	0.039	0.00112	0.008	0.02412	0.996
Ferulic acid	0.053	0.00266	-0.078	0.06154	0.992
Syringaldehyde	0.010	0.00026	-0.008	0.00537	0.998
Syringic acid	0.001	0.00003	-0.001	0.00072	0.998
Sinapaldehyde	0.049	0.00180	-0.054	0.03922	0.996

Table S13. Concentration of vanillin, vanillic acid, coniferyl aldehyde, ferulic acid, syringaldehyde, syringic acid and sinapaldehyde obtained in the investigated extracts. Being a softwood, fir wood does not contain syringaldehyde, syringic acid and sinapaldehyde. (<LOD): the concentration was below the LOD of method.

	**Vanillin**					
	**Ethanol**		**Acetone**		**Ethyl lactate**	
	**Oak (µg/g)**	**Fir (µg/g)**	**Oak (µg/g)**	**Fir (µg/g)**	**Oak (µg/g)**	**Fir (µg/g)**
**CP (average)**	3.0 ± 1	9.0 ± 3	16.0 ± 6	23.0 ± 2	8.0 ± 2	15.0 ± 6
**Experiment 1**	6.0	<LOD	6.6	<LOD	<LOD	6.2
**Experiment 2**	11.0	12.5	9.5	<LOD	7.3	<LOD
**Experiment 3**	1.9	4.8	<LOD	<LOD	<LOD	<LOD
**Experiment 4**	7.0	16.9	<LOD	<LOD	<LOD	3.0
**Experiment 5**	14.3	<LOD	5.6	17.6	5.3	3.3
**Experiment 6**	14.0	42.5	16.3	<LOD	29.7	37.2
**Experiment 7**	83.5	7.2	5.5	8.7	<LOD	6.3
**Experiment 8**	10.8	17.0	5.9	7.9	4.7	12.6

	**Vanillic acid**					
	**Ethanol**		**Acetone**		**Ethyl lactate**	
	**Oak (µg/g)**	**Fir (µg/g)**	**Oak (µg/g)**	**Fir (µg/g)**	**Oak (µg/g)**	**Fir (µg/g)**
**CP (average)**	3.0 ± 1	5.0 ± 2	6.0 ± 2	9.0 ± 2	6.0 ± 2	20.0 ± 3
**Experiment 1**	4.0	<LOD	<LOD	<LOD	<LOD	<LOD
**Experiment 2**	4.5	22.2	3.5	0.9	8.2	<LOD
**Experiment 3**	<LOD	<LOD	<LOD	<LOD	<LOD	<LOD
**Experiment 4**	6.2	39.6	<LOD	<LOD	8.8	<LOD
**Experiment 5**	22.6	25.7	3.7	18.8	7.4	15.4
**Experiment 6**	18.6	22.1	6.3	<LOD	5.4	32.5
**Experiment 7**	9.7	18.2	<LOD	5.0	4.4	12.7
**Experiment 8**	3.4	37.9	2.0	4.7	8.2	11.3

	**Coniferyl aldehyde**					
	**Ethanol**		**Acetone**		**Ethyl lactate**	
	**Oak (µg/g)**	**Fir (µg/g)**	**Oak (µg/g)**	**Fir (µg/g)**	**Oak (µg/g)**	**Fir (µg/g)**
**CP (average)**	12.0 ± 6	14.0 ± 7	8.0 ± 1	17.0 ± 3	3.0 ± 2	7.0 ± 5
**Experiment 1**	<LOD	4.7	3.1	<LOD	<LOD	<LOD
**Experiment 2**	9.8	22.6	8.1	4.1	<LOD	<LOD
**Experiment 3**	<LOD	2.3	<LOD	<LOD	<LOD	<LOD
**Experiment 4**	3.9	16.1	<LOD	<LOD	<LOD	<LOD
**Experiment 5**	6.8	12.8	4.2	4.2	4.3	12.7
**Experiment 6**	6.8	24.5	9.2	<LOD	7.5	18.5
**Experiment 7**	13.4	15.2	2.3	<LOD	2.3	6.9
**Experiment 8**	<LOD	17.6	7.8	16.2	5.1	9.2

	**Ferulic acid**					
	**Ethanol**		**Acetone**		**Ethyl lactate**	
	**Oak (µg/g)**	**Fir (µg/g)**	**Oak (µg/g)**	**Fir (µg/g)**	**Oak (µg/g)**	**Fir (µg/g)**
**CP (average)**	7.0 ± 3	8.0 ± 4	3.3 ± 0.2	5.9 ± 0.03	7.0 ± 2	7.0 ± 2
**Experiment 1**	5.9	5.9	9.1	<LOD	4.7	<LOD
**Experiment 2**	6.0	6.0	9.3	8.9	8.9	<LOD
**Experiment 3**	6.0	5.9	<LOD	<LOD	<LOD	<LOD

**Experiment 4**	6.0	5.9	12.0	<LOD	8.9	<LOD
**Experiment 5**	9.4	<LOD	9.4	8.9	6.0	6.0
**Experiment 6**	9.0	9.0	9.3	8.9	6.2	6.0
**Experiment 7**	6.2	6.0	8.9	8.9	8.9	8.9
**Experiment 8**	6.3	5.9	9.1	8.9	8.9	9.0

	**Syringaldehyde**		
	**Ethanol**	**Acetone**	**Ethyl lactate**
	**Oak (µg/g)**	**Oak (µg/g)**	**Oak (µg/g)**
**CP (average)**	25.0 ± 10	35.0 ± 5	28.5 ± 0.6
**Experiment 1**	12.4	16.5	5.6
**Experiment 2**	38.3	37.6	11.6
**Experiment 3**	5.5	6.2	4.7
**Experiment 4**	15.4	7.9	5.6
**Experiment 5**	35.3	21.9	21.9
**Experiment 6**	25.4	36.1	42.9
**Experiment 7**	48.8	14.0	16.0
**Experiment 8**	22.7	31.7	36.5

	**Syringic acid**		
	**Ethanol**	**Acetone**	**Ethyl lactate**
	**Oak (µg/g)**	**Oak (µg/g)**	**Oak (µg/g)**
**CP (average)**	25.0 ± 1	35.0 ± 5	76.0 ± 2
**Experiment 1**	5.3	14.6	7.6
**Experiment 2**	44.3	18.2	16.4
**Experiment 3**	8.7	<LOD	4.2
**Experiment 4**	22.2	<LOD	5.0
**Experiment 5**	48.2	31.1	24.2
**Experiment 6**	43.4	23.2	28.9
**Experiment 7**	28.0	7.7	17.8
**Experiment 8**	65.8	17.1	19.4

	**Sinapaldehyde**		
	**Ethanol**	**Acetone**	**Ethyl lactate**
	**Oak (µg/g)**	**Oak (µg/g)**	**Oak (µg/g)**
**CP (average)**	12.1 ± 0.1	9.2 ± 0.9	8.0 ± 2
**Experiment 1**	5.3	7.9	6.6
**Experiment 2**	10.4	12.1	<LOD
**Experiment 3**	5.0	8.8	<LOD
**Experiment 4**	7.3	8.9	<LOD
**Experiment 5**	15.8	11.9	8.3
**Experiment 6**	11.1	14.1	11.2
**Experiment 7**	14.1	7.4	7.5
**Experiment 8**	8.5	11.9	7.5

Figure S2. Coefficient plots and respective contour plots showing the influence of the investigated factors on the sinapaldehyde concentration (µg/g) of oak and fir wood.

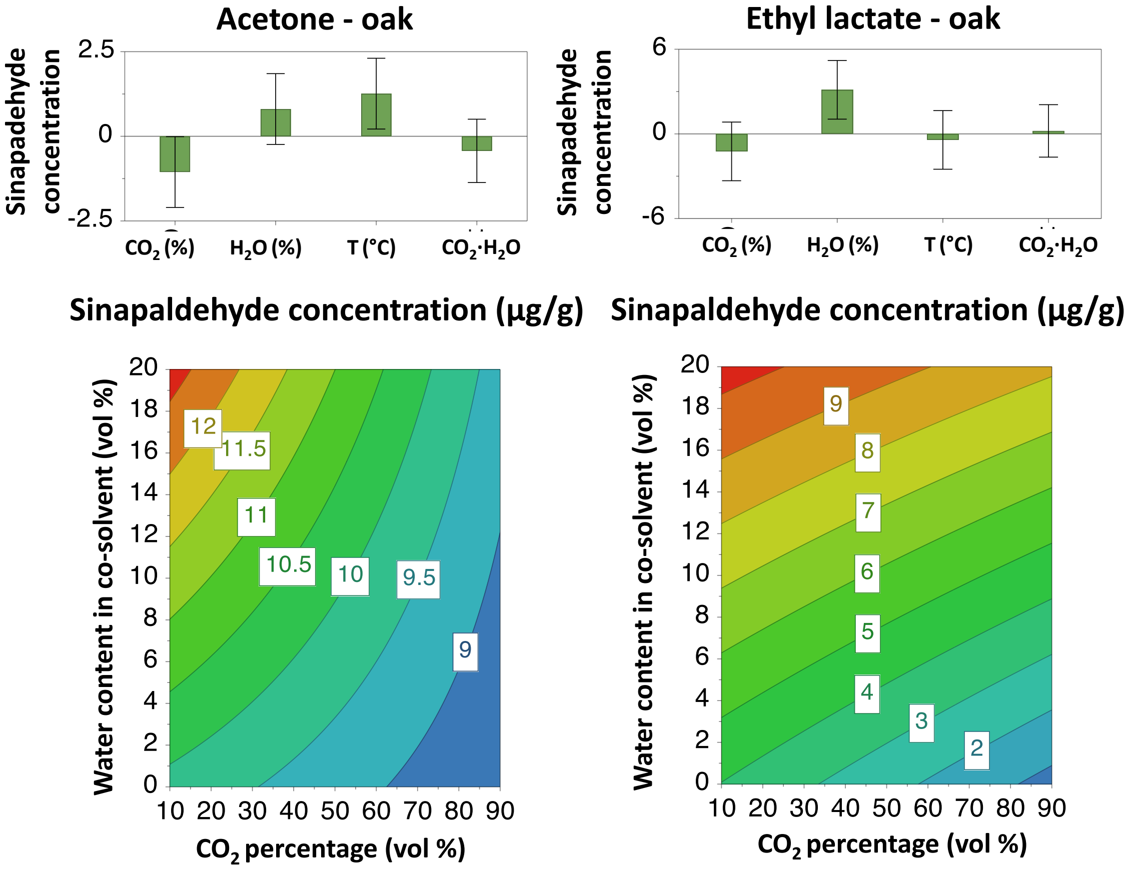

Figure S2 - Coefficient plots from the interaction model with a full factorial design showing the influence of the investigated factors and two-factor interactions on the sinapaldehyde concentration and contour plots showing the influence of the CO_2 percentage and water content in the co-solvent on the sinapaldehyde concentration of oak wood using acetone and ethyl lactate as co-solvent. CO_2 (%): CO_2 percentage; H_2O (%): water content in co-solvent. Temperature was 60 °C and pressure 350 bar.

Figure S3. Base peak ion chromatogram of the lignin extracted from oak wood obtained with UHPSFC/ESI-HRMS.

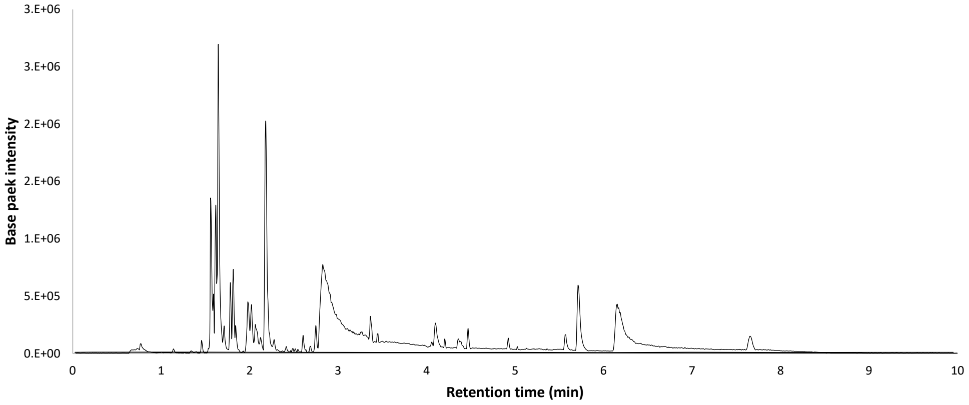

Figure S4. Loading plot of the KMD-PCA-QDA classification model for lignin dimers showing the latent variables (LVs) 1 and 3. #C = number of carbon atoms, #H = number of hydrogen atoms, #O = number of oxygen atoms.

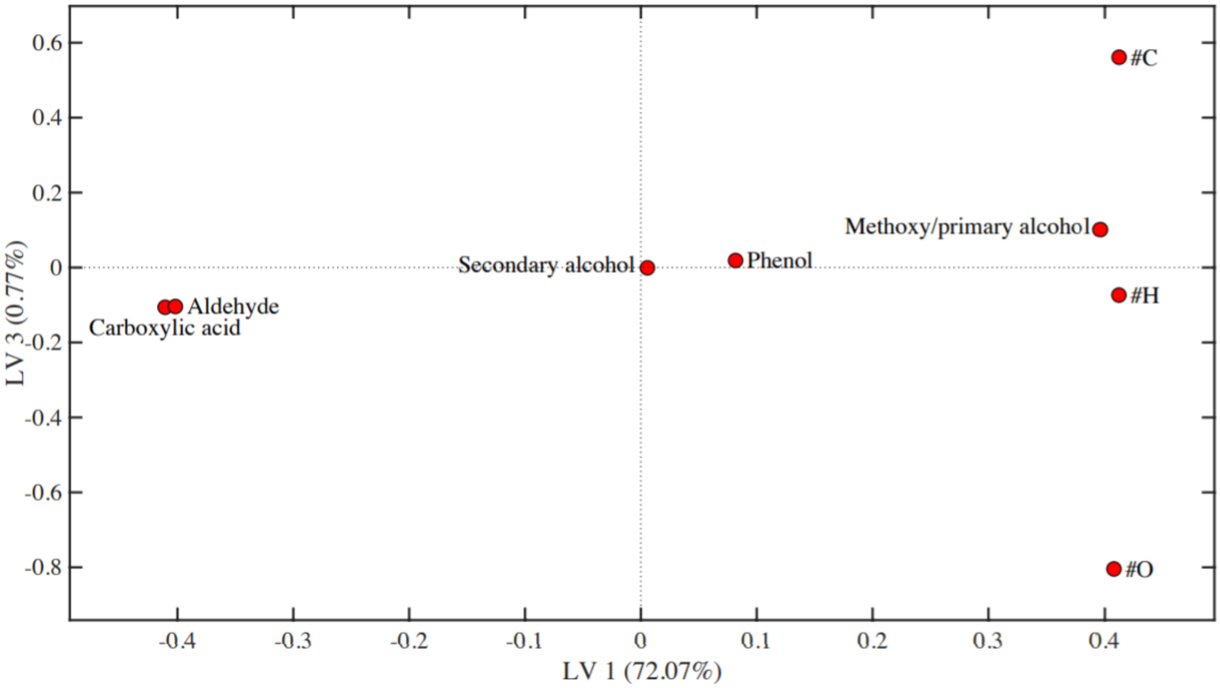

Supplement: RA-013-D3RA01873C-s001 [file RA-013-D3RA01873C-s001.pdf]
